# Evaluation of the marginal adaptation and debonding strength of two types of CAD-CAM implant-supported cement-retained crowns

**DOI:** 10.1186/s12903-023-03711-7

**Published:** 2023-12-05

**Authors:** Nada Ahmed Ramzy, Amir Shoukry Azer, Mohamed Moataz Khamis

**Affiliations:** 1 Faculty of Dentistry, Alexandria University, Alexandria, 8, Abbas Alhalawany Street Ibrahemia, Egypt; 2 Department of Conservative Dentistry, Faculty of Dentistry, Alexandria University, Alexandria, Egypt; 3 Department of Prosthodontics, Faculty of Dentistry, Alexandria University, Alexandria, Egypt

**Keywords:** Abutment, Ceramics, Crown, Implant, Lithium disilicate, Polymers, Composite, Marginal fit, Adaptation, Margin, Debonding

## Abstract

**Background:**

In-vitro data from a clinically well-known lithium disilicate ceramic reference was used to assess the expected performance of resin-based materials in implant dentistry. The purpose of the study was to compare the bond strength and marginal adaptation of nano-ceramic hybrid composite crowns cemented to stock cement-retained abutments to lithium disilicate crowns.

**Methods:**

Twenty abutment analogs were embedded into auto-polymerizing acrylic resin blocks. The blocks were divided into 2 groups according to the restorative crown material. The 2 groups were divided as follows: Resin nano-ceramic group and lithium disilicate group. Abutment analogs in both groups were scanned using a laboratory scanner, and the restorations were designed, manufactured, and cemented with resin cement over the corresponding group. All samples were tested for marginal adaptation and bond strength after storage for 24 hours at 37 °C in 100% humidity. Data were collected, tabulated, and statistically analysed using the appropriate tests. Normality was checked using Shapiro Wilk test and Q-Q plots. Data were normally distributed. Variables were presented using mean, 95% Confidence Interval (CI) and standard deviation in addition to median and Inter Quartile Range (IQR). Differences between groups regarding debonding forces was assessed using independent t test. Two Way ANOVA was performed to assess the effect of material and bonding on marginal gap. All tests were two tailed and *p* value was set at < 0.05.

**Results:**

Marginal gap and debonding force values were significantly different according to the type of material used (*P* < .05). Resin nano-ceramic crowns presented lower marginal gap values before (20.80 ± 8.87 μm) and after (52.11 ± 22.92 μm) bonding than lithium disilicate crowns. The debonding force value for resin nano-ceramic crowns (284.30 ± 26.44 N) was significantly higher than that for lithium disilicate crowns (253.30 ± 33.26 N). Adhesive failure mode was detected in all the specimens in both groups.

**Conclusions:**

The type of material used for implant-supported cement-retained crowns had a statistically significant effect on marginal adaptation and bond strength. Resin nano-ceramic implant-supported cement-retained crowns had better marginal adaptation and higher bond strength than those manufactured using lithium disilicate.

## Background

All-ceramic computer aided design, computer aided manufacture (CAD-CAM) materials are increasingly being used for implant-supported restorations, particularly in the dental arch’s aesthetic area. One of the most widely used CAD-CAM materials is lithium disilicate ceramics. This high-esthetic and high-strength ceramic material has proven its clinical longevity and showed good clinical results [1–5]. Nevertheless, CAD-CAM lithium disilicate ceramics require time-consuming crystallization after milling, polishing and/or glazing.

Hybrid CAD-CAM materials represent a feasible substitute for aesthetic dental restorative materials. They have sufficient fatigue resistance to withstand the forces of mastication, according to the literature [6]. As in resin hybrid nano-ceramic crowns, firing processes are not required and polishing is performed by using abrasive disks [7]. They can be easily stained and repaired by direct composites [8]. Moreover, they are not brittle and are relevant to the opposing dentition [9].

Resin nano-ceramic hybrid materials consist of a resin type material with a blend of 86% filled nano-ceramics in a “polymer network”. This material combination offers enhanced longevity and might be an alternative for implant-supported restorations [10, 11]. So far, the effect of such a structural modification on marginal adaptation and debonding forces has not yet been thoroughly investigated as an implant-supported restoration.

The success and durability of any restoration are related to 3 main criteria; strength, fit and esthetics [12]. Previous studies have shown that the marginal adaptation of CAD-CAM restorations is material dependent [13–16]. A suitable marginal adaptation ensures minimal cement film thickness [15] and prevents micro leakage that could lead to prosthesis failure [13, 17]. It is reported that marginal gap of less than 120 or 150 μm is considered an acceptable clinical goal [18, 19]. Regarding the margin location on the samples of this study, it was between the restoration margin and the abutment finish line. The recommendation of margin placement clinically for cement retained restorations will depend on esthetic demands. If esthetics is not critical, supragingival placement of the finish line is recommended. While if esthetics is critical, a 1 mm subgingival finish line is recommended to be able to properly remove excess cement. According to a previous research, the restoration margin’s placement is highly correlated with cement that remains undetected after cleaning. Clinically it was recommended that locating a margin 2 mm below the gingival level is risky [20].

Recent literatures spotlight the properties of machinable lithium disilicate. Bergamo et al [21] evaluated the effect of shrinkage of lithium disilicate during the crystallization process. It was concluded that shrinkage does not affect the margin fit.

In vitro evaluations reported mean values between 11 and 67.4 μm for the marginal gaps of metal–ceramic crowns cemented to implant abutments [22, 23], and between 65.9 and 168 μm for all-ceramic crowns cemented to metal implant abutments [24, 25]. Accordingly, there are no enough data regarding the marginal fit of implant-supported nano-ceramic resin crowns on titanium abutments.

Another functional parameter, aside from the marginal adaptation, is the bond strength. A successful long-term bonding of esthetic restorations to titanium abutments in the oral environment is crucial for restoration longevity.

Saber et al [26] stated that the retention of narrow platform cement-retained restorations is influenced by the wall height but not in same manner as wide platform. Restorations of narrow-platform size with longer abutment exhibited higher tensile resistance to dislodgement. Rismanchian et al [27] stated that using nano or micro airborne abrasive particles is an efficient way for increasing bond strengths significantly, but it seems that micro airborne abrasive particles was more effective.

The need for retrievability of cement retained restorations is usually for 2 reasons. One to remove excess cement which would be easily removed if the restoration margins were supragingival or 1 mm subgingival as recommended in several studies [20, 28]. If deep subgingival finish lines have to be used, a screw access channel should be created on the occlusal surface of the cement retained restoration to allow its retrievability to remove excess cement [29]. In both those cases it is imperative to lute the restorations as best as possible to avoid restoration debonding.

Resin nano-ceramic material is already being clinically used, even though no clinical data on its long-term success as an implant-supported restoration is yet available. However, preliminary studies have shown promising results in terms of its strength, durability, and biocompatibility. Further research is needed to fully understand its potential as a reliable alternative to traditional all-ceramic CAD-CAM restorative dental materials.

The aim of the current study was to evaluate the marginal adaptation and bond strength of resin nano-ceramic posterior cement-retained implant-supported crowns compared to lithium disilicate ones. The null hypothesis was that there will be no significant difference in marginal adaptation and bond strength between the resin nano-ceramic and lithium disilicate implant-supported cement-retained crowns.

## Methods

**Fig. 1:**
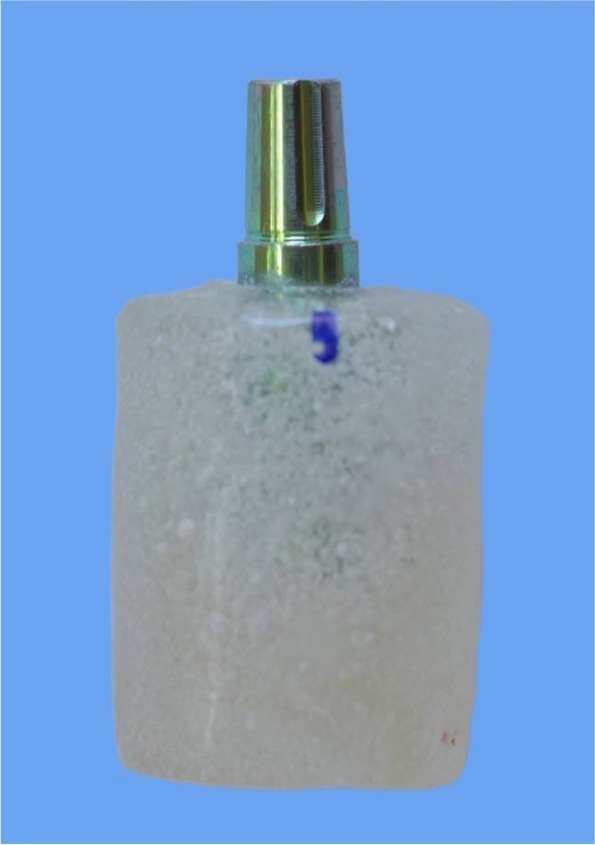
Universal abutment analogs embedded in acrylic resin blocks

Twenty abutment laboratory analogs (Neodent; Straumann Group, Brazil) with a diameter of 4.5 mm and a height of 6 mm were vertically embedded into auto-polymerizing acrylic resin blocks fabricated in a specially designed copper with a diameter of 2 cm cylindrical mold (Fig. 1). A surveyor (Ney surveyor; Dentsply Sirona) was used to standardize the long axis alignment of the implant analogs for the pull-off testing.

The resin blocks were randomly divided into 2 groups (*n* = 10 each) by using a computer-generated list of random numbers (www.randomizer.org). According to the crown material used, the 2 groups were divided as follows: Resin nano-ceramic group (Grandio bloc; Voco, Germany) and lithium disilicate group (IPS e.max CAD; Ivoclar Vivadent, Liechtenstein).

**Fig. 2:**
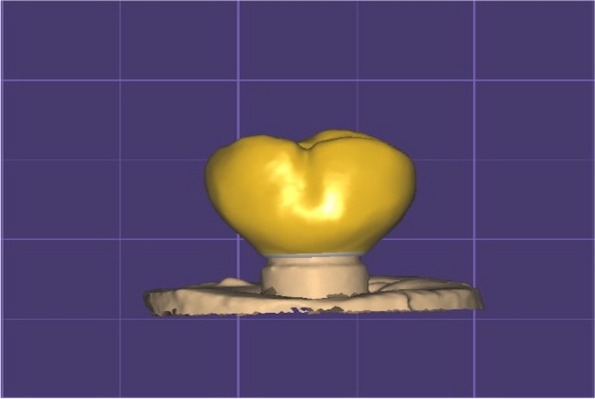
A full contour maxillary first molar using exocad software

A laboratory scanner (Ceramil Map 400; Amann Girrbach, Austria) was used to fabricate the crowns for each group. A full contour maxillary first molar was designed by using a CAD software program (exocad software; exocad GmbH, Germany) in standard tessellation language (STL) format (Fig. 2). Similar crown parameters and dimensions were used for all crowns in both groups. A milling machine (CEREC MC X5; Dentsply Sirona, USA) was used to mill the crowns of the 2 groups. Standardization was done by using the same crown design and parameters already selected through the CAD software, which was used for all specimens and subsequently milled, thus all specimens had same crown size and design. Crowns for the resin nanoceramic group were just polished, while crowns for the lithium disilicate group were dried, glazing material added, and then crystallised and glazed in a ceramic furnace (Programat P310; Ivoclar AG, Liechtenstein).

Prior to bonding, the marginal gap of the crowns was evaluated by using a stereomicroscope (Zeiss Stereomicroscope, Germany) under × 50 magnification [30], and photographed and analyzed by using an image analysis software (Olympus DP2-SAL; Olympus Corp, Japan) to evaluate the fit accuracy of the crowns. Six points were identified on the buccal side (mesiobuccal, midbuccal and distobuccal) and lingual side (mesiolingual, midlingual and distolingual) of each crown as reference points for the measurement of marginal gap values.

**Fig. 3:**
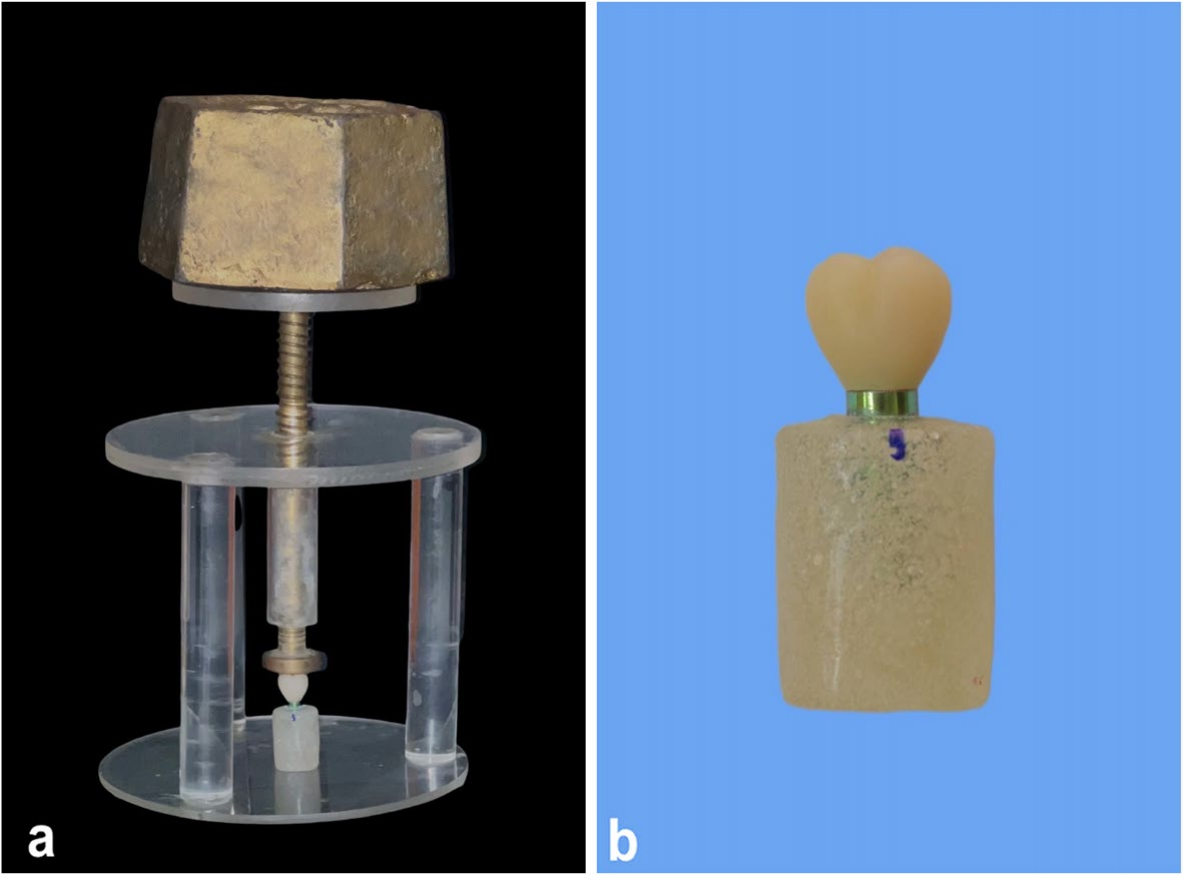
**a** Static load device, **b** Removal of excess cement to ensure clear margin, crowns seated on corresponding abutment

Only the crowns for the lithium disilicate group were etched with 9.5% hydrofluoric acid (Buffered Hydrofluoric Acid Gel, Bisco, USA) for 20 seconds, followed by rinsing and air drying. A silane coupling agent (porcelain primer; Bisco, USA) was applied to all crowns with 2 layers then drying. This was followed by applying a resin cement (Duo-Link, Universal adhesive resin cement, Bisco, USA) to cement all the crowns according to the manufacturer’s instructions. A 2-kg static load was applied to the crowns with a static load device to ensure standardization and complete seating during the setting of the resin cement (Fig. 3a) [31]. A clear margin was ensured by the removal of excess cement (Fig. 3b).

**Fig. 4:**
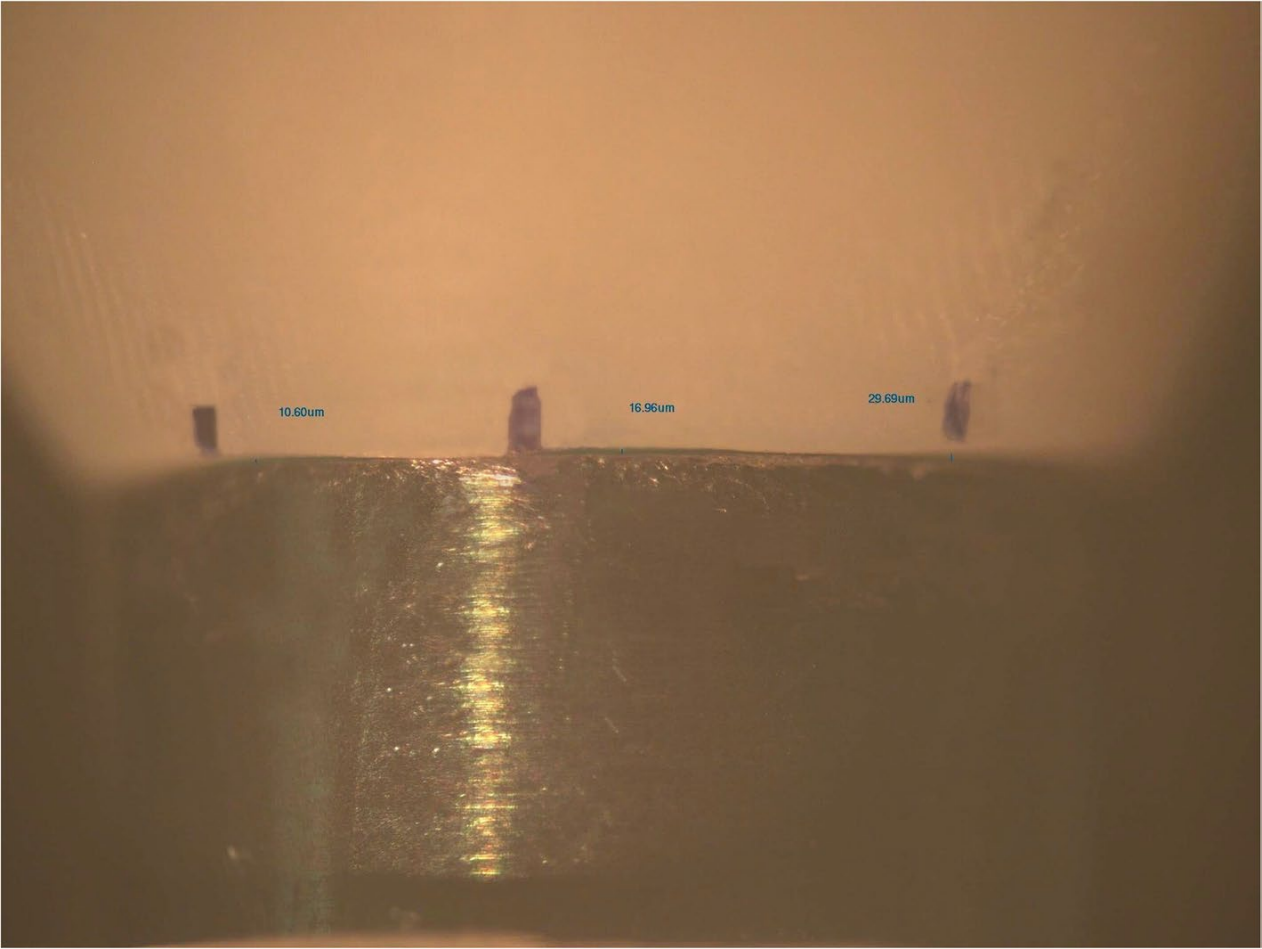
Measuring points after bonding under magnification × 50

After bonding, the marginal gap of the crowns was reevaluated by using the same procedure (Fig. 4). All crowns were stored at 37 °C in 100% humidity for 24 hours [31].

**Fig. 5:**
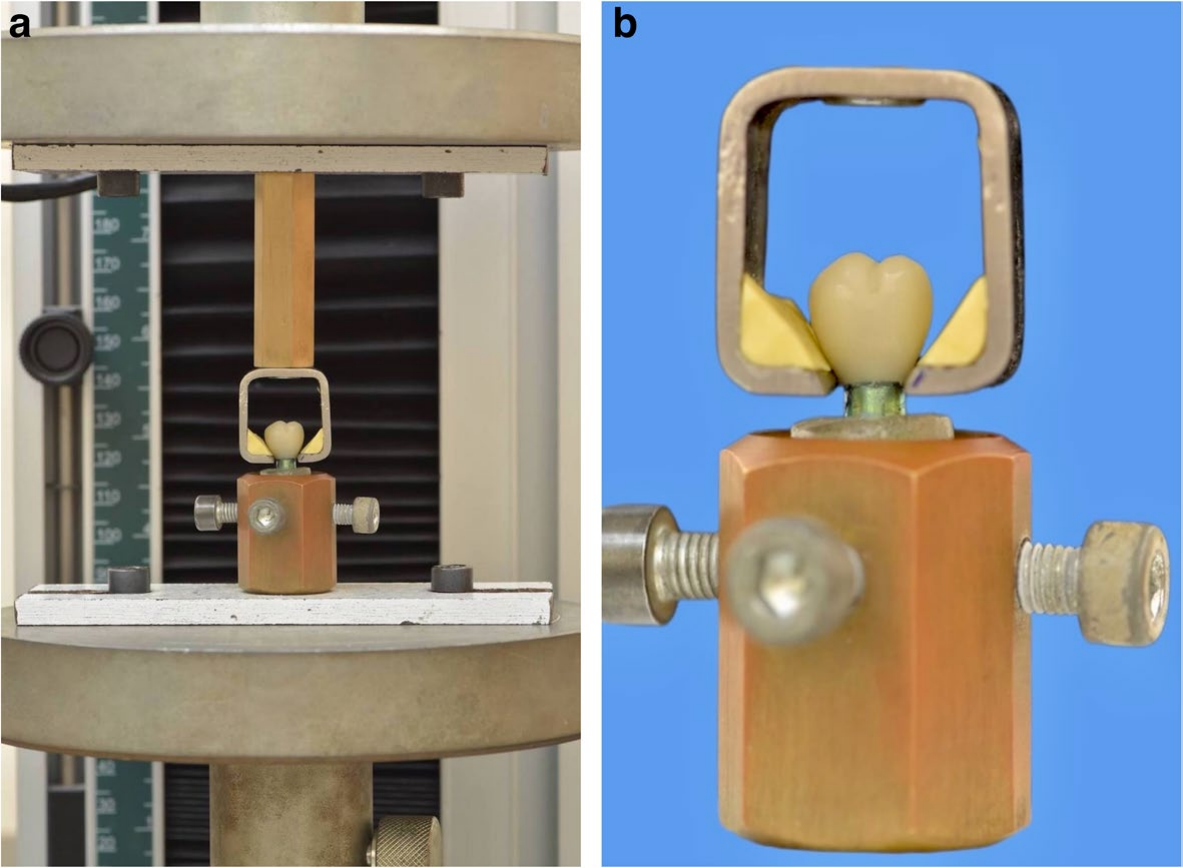
**a** Specimen transferred and mounted on the universal testing machine, **b** Attachment designed for pulling the samples lined with a shock absorbing material

All specimens were individually transferred and mounted to a universal testing machine (5st, tinius olsen, England) at a 0.5-mm/min cross head speed. The debonding force required to debond each restoration from its abutment was measured in newtons by using a universal testing machine (Fig. 5a).

The de-bonded crowns were examined at 25× magnification using an optical stereomicroscope (Zeiss Stereomicroscope, Germany) for signs of gingival margin defects. All defects or irregularities were registered and graded on a scale of 1–3, according to severity, on a Likert scale [32, 33] as follows: Grade 1: Optimal margins without flaws; Grade 2: Multiple chips, or uneven margins; Grade 3: Large defects visible without a microscope.

Data were collected and statistically analyzed with a statistical software program (IBM SPSS Statistics for Windows, v23.0; IBM Corp) [34]. Normality was checked for all variables by using Shapiro Wilk test and Q-Q plots. Data were normally distributed. Variables were presented using mean, 95% Confidence Interval (CI) and standard deviation in addition to median and Inter Quartile Range (IQR). Differences between groups regarding debonding forces was assessed using independent t test. Two Way ANOVA was performed to assess the effect of material and bonding on marginal gap. All tests were 2 tailed (*P* < .05).

## Results

The mean vertical marginal gap in (μm) and standard deviation for the 2 groups are shown in (Table 1). The vertical marginal gap results were measured. The results showed that the highest mean values for the vertical marginal gap were reported for the lithium disilicate group before (52.11 ± 22.92 μm) and after (79.23 ± 21.77 μm) bonding, while the lowest mean value was reported for the resin nanoceramic group before (20.80 ± 8.87 μm) and after (26.52 ± 10.96 μm) bonding. The Increase in marginal gap values after bonding was not significant in the resin nanoceramic group (*P* = .078), while it was statistically significant in the lithium disilicate group (*P* < .0001) as illustrated in Table 1.

**Table 1:** Comparison of marginal gap before and after bonding between the study groups (gap sizes presented in micron)

		Resin nanoceramic Group (*n* = 10)	Lithium disilicate Group (*n* = 10)
Before Bonding	Mean ± SD	20.80 ± 8.87	52.11 ± 22.92
	95% CI for mean	16.65, 24.95	41.39, 62.84
	Median (IQR)	19.85 (15.02)	52.66 (36.41)
After bonding	Mean ± SD	26.52 ± 10.96	79.23 ± 21.77
	95% CI for mean	21.39, 31.65	69.04, 89.42
	Median (IQR)	28.98 (17.50)	74.60 (45.42)
*P* value		0.078	< 0.0001*

*Statistically significant difference at *P* ≤ .05, 95% CI: Confidence Interval

Two-way ANOVA showed that material type and bonding had a significant effect on the marginal gap of the crowns (*P < .0001*). Furthermore, the effect of their interaction was also statistically significant (*P* = .007). Material type had the highest significant effect size on the marginal adaptation (partial eta squared value 0.608 η) (Table 2).

**Table 2:** Two Way ANOVA assessing the effect of material and bonding on marginal adaptation

	Mean square	*p* value	Partial Eta Squared (η)
Material	35,294.702	< 0.0001*	0.608
Bonding	5391.835	< 0.0001*	0.192
Material x Bonding	2289.336	0.007*	0.091

*Statistically significant difference at *P* ≤ .05

The means and standard deviations for tensile debonding force values recorded in the 2 groups are presented in Table 3. Tensile debonding force values were significantly different according to the type of crown material used (*P* = .033). Resin nanoceramic group showed higher mean debonding force values (284.30 ± 26.44 N) when compared to lithium disilicate group (253.30 ± 33.26 N) as presented in Table 3.

**Table 3:** Comparison of debonding forces between the study groups

	Resin nanoceramic Group (*n* = 10)	Lithium disilicate Group (*n* = 10)	Test (*P*)
Mean ± SD	284.30 ± 26.44	253.30 ± 33.26	2.307 (0.033*)
95% CI for mean	265.39, 303.21	229.50, 277.10	
Median (IQR)	283.50 (50.00)	247.00 (62.00)	

*Statistically significant difference at *P* ≤ .05, 95% CI: Confidence Interval

**Fig. 6:**
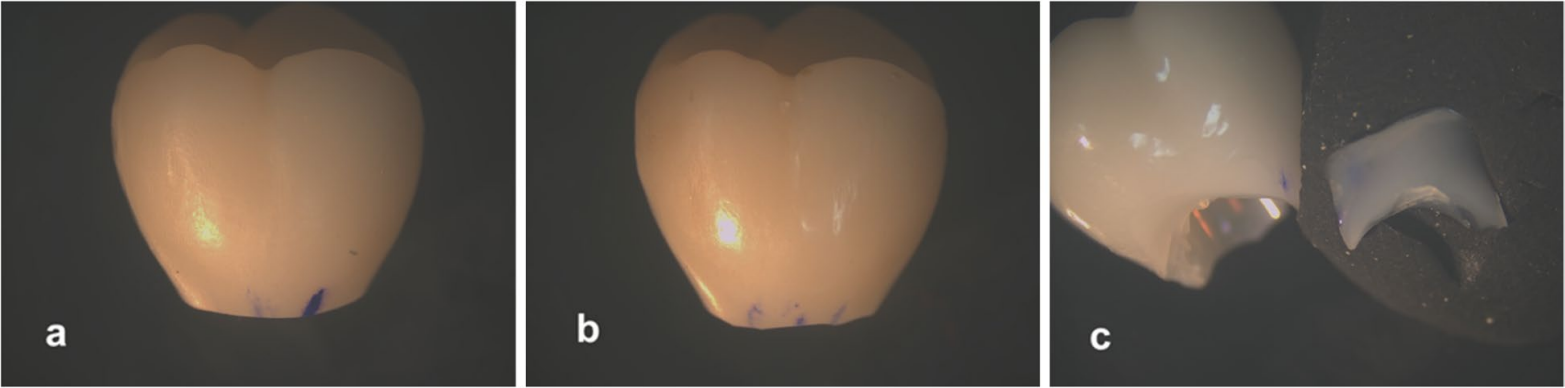
Gingival margin defects on a Likert scale as follows: **a** Grade 1. Optimal margins without flaws; **b** Grade 2. Multiple chips, or uneven margins; **c** Grade 3. Large defects visible without a microscope

Adhesive failure mode was detected in all the specimens in both groups. Crowns for resin nanoceramic group displayed grade 1 and 2 marginal defects, whereas crowns for lithium disilicate group displayed only grade 3 marginal defects resulting from debonding forces (Fig. 6).

## Discussion

The null hypothesis was rejected since there was a statistically significant difference between the values of the two tested groups in marginal adaptation and bond strength between the resin nano-ceramic and lithium disilicate CAD-CAM implant-supported cement retained crowns.

Early in-vivo performances of resin nano-ceramic CAD-CAM restorations are encouraging, leading to the availability of a wide range of new tooth-colored resin nano-ceramic blocks. The drawbacks of ceramic restorations have stimulated the search for alternatives. The advantage of using resin-based materials with their obvious resiliency in comparison to ceramic based materials for constructing restorations over dental implants is their shock absorbing qualities, damping excessive forces transmitted to implants [10, 11]. Although it seems logical to recommend the use of those materials over implants, research and studies should be performed to evaluate the various characteristics of those materials to validate their performance and use over implants.

This study examined the marginal gap of 2 CAD-CAM materials before and after bonding to detect their primary precision. The gap was measured before bonding to eliminate any variables that could complicate obtaining proper information about the precision of the marginal gap, as noted by other authors [17, 35, 36].

Many studies favored visual assessment of vertical marginal defect with a stereomicroscope over a destructive approach or replica technique [37–40]. The present study used a stereomicroscope to perform microscopic analysis at × 50 magnification [26]. Static pressure was used during bonding to ensure uniform and complete seating of the crowns (Fig. 3a). Six measurements were made for each crown before and after bonding (total 240 measurements). This number was enough to give a consistent estimate for the gap size [41].

This study found that resin nano-ceramic implant-supported crowns had a smaller mean marginal gap value than lithium disilicate crowns before and after bonding. The mean marginal gap values before bonding ranged between 20.80 μm for resin nanoceramic group and 52.11 μm for lithium disilicate group (Table 1). This is in accordance with other studies [7, 37] that reported that resin-based blocks had visibly smoother margins and superior marginal adaptation compared to lithium disilicate. The 2 CAD materials have different microstructures and mechanical properties. Resin nanoceramics are less brittle than lithium disilicate, resulting in better milling quality and accuracy. Lithium disilicate CAD is also milled in a precrystallized stage, with low strength against chipping giving a better advantage of the resin nanoceramic material.

The differences in the values between the 2 groups before bonding can be attributed to the effect of the different fabrication procedure on the 2 studied materials. Gold et al [42] found that lithium disilicate CAD-CAM crowns experienced an increase in marginal gap following crystallization. Besides, Mounajjed et al [43] reported that lithium disilicate restorations fabricated with CAD-CAM techniques have larger marginal gaps than those fabricated with press techniques, which are within a clinically acceptable range.

Previous studies have reported that marginal gap values increased after bonding [44]. In the present study, crowns of the resin nanoceramic group had a better marginal fit than those for the lithium disilicate group, both before and after bonding. Bonding significantly increased the marginal gap values for lithium disilicate group only (*P* < .0001).

Two Way ANOVA was performed to assess the effect of material and bonding on marginal gap. It was found that the material type used had the highest partial eta squared (0.608), while bonding had less significant effect. Therefore, it can be concluded that the type of material used for dental restorations has a greater impact on the marginal gap than the bonding process.

The design of debonding device used with the Universal Testing Machine (5st, tinius olsen, England) was made specially for the present study, similar to Rohr et al [45], Khalifa et al [46] and Ibrahim et al. [47] It was constructed from a long cylinder made of copper and square stainless steel frame to accommodate the crown, with rubber putty inside the device as a shock absorbing material to prevent early fracture of crowns (Fig. 5b).

A reliable bond between materials is crucial for successful cemented solutions, ensuring structural integrity and preventing potential failure or separation [22]. Based on the current study results, crown retention was significantly affected by the material used for restoration construction, with resin nanoceramic group (284.30 N) showing higher mean debonding forces than lithium disilicate group (253.30 N). The difference between the 2 groups was statistically significant (*P* = 0.033). This is due to the fact that resin nano-ceramic is chemically bonded to the resin cement used, while lithium disilicate depends on micro-mechanical interlocking.

The low retention values in both groups might be a result of the smooth surface of titanium abutments, which were not modified or surface treated [48]. These abutments are simple to use and offer predictable retention and fit of the crown [49]. Adhesion failure was the mode of failure for both groups, occurring at the interface between the abutment surface and cement layer also probably due to the smooth abutment surface.

The behavior of crown margins after the application of debonding forces was detected under × 25 magnification. Results showed that the lithium disilicate crowns were more brittle in thin sections than the nano-ceramic resin hybrid crowns, that might affect their long-term durability and resistance to fracture. These findings suggest that the choice between the 2 materials should be based on the specific clinical situation and patient preferences.

This study had some limitations as the crowns were tested under in vitro conditions, which might be different from clinical conditions. Additionally, aging processes such as thermocycling and cyclic loading were not performed. As present study tried to focus on maximum bonding values and marginal gap measurements without external factors. Since it is well documented that such procedures will definitely reduce the bond strength as mentioned by several previous studies [50, 51]. Further invivo studies are required.

## Conclusions

Based on the findings of this in vitro study, results of this preliminary study showed that resin nano-ceramic implant-supported cement-retained crowns had better marginal adaptation than those fabricated by using lithium disilicate. Moreover, bond strength of resin nano-ceramic implant-supported cement-retained crowns was significantly higher than that of lithium disilicate.

## Data Availability

The raw data of the present study is available at: 10.6084/m9.figshare.23937585.

## Abbreviations

CAD: Computer aided designing
CAM: Computer aided manufacturing
CI: Confidence Interval
Fig.: Figure
SD: Standard Deviation
